# A Dynamic Bioinspired Neural Network Based Real-Time Path Planning Method for Autonomous Underwater Vehicles

**DOI:** 10.1155/2017/9269742

**Published:** 2017-02-01

**Authors:** Jianjun Ni, Liuying Wu, Pengfei Shi, Simon X. Yang

**Affiliations:** ^1^College of IOT Engineering, Hohai University, Changzhou 213022, China; ^2^Changzhou Key Laboratory of Special Robot and Intelligent Technology, Hohai University, Changzhou 213022, China; ^3^Advanced Robotics and Intelligent Systems (ARIS) Laboratory, School of Engineering, University of Guelph, Guelph, ON, Canada N1G 2W1

## Abstract

Real-time path planning for autonomous underwater vehicle (AUV) is a very difficult and challenging task. Bioinspired neural network (BINN) has been used to deal with this problem for its many distinct advantages: that is, no learning process is needed and realization is also easy. However, there are some shortcomings when BINN is applied to AUV path planning in a three-dimensional (3D) unknown environment, including complex computing problem when the environment is very large and repeated path problem when the size of obstacles is bigger than the detection range of sensors. To deal with these problems, an improved dynamic BINN is proposed in this paper. In this proposed method, the AUV is regarded as the core of the BINN and the size of the BINN is based on the detection range of sensors. Then the BINN will move with the AUV and the computing could be reduced. A virtual target is proposed in the path planning method to ensure that the AUV can move to the real target effectively and avoid big-size obstacles automatically. Furthermore, a target attractor concept is introduced to improve the computing efficiency of neural activities. Finally, some experiments are conducted under various 3D underwater environments. The experimental results show that the proposed BINN based method can deal with the real-time path planning problem for AUV efficiently.

## 1. Introduction

Autonomous underwater vehicle (AUV) has attracted much attention in recent years, due to its application in both commercial and military fields [1, 2]. AUVs can be used for searching missing airplanes and ships wreckage, catching underwater evaders, maritime rescuing, mine countermeasures, antisubmarine warfare, and so on [3–5]. In these applications of AUVs, many issues should be efficiently solved, such as localization, path planning, and target detection [6–8]. Among these issues, real-time path planning is very basic and necessary when a single AUV or a multi-AUV system executes a task in complex underwater environments [9–11]. The task of real-time path planning in this study is to find an optimal or suboptimal collision-free path from the initial position to the target location in an underwater environment quickly and efficiently, which is a difficult and challenging task because of the complexity of underwater environments.

A lot of research work has been done on the path planning problem for AUVs. For example, Pêtrès et al. [12] presented a novel fast marching based approach which extracted a continuous path from the discrete environment representation and took the underwater current into account. Zhu et al. [13] applied the Dempster-Shafer inference rule to fuse the readings of the ultrasonic sensor into a map and combined the map and bioinspired neural network to plan a short and smooth path for an AUV. Yilmaz et al. [14] defined the path planning problem as an optimization framework and presented a mixed integer linear programming based method. Those methods introduced above are all tested in two-dimensional (2D) environments, which does not meet the actual requirement of AUV path planning.

Recently, more and more researchers focus on the path planning problem in three-dimensional (3D) underwater environments. Hu et al. [15] developed a vision-based autonomous robotic fish which is capable of 3D locomotion, by using a control law with an attractive force toward a target and a repulsive force against obstacles. Aghababa [16] applied five evolutionary algorithms to solve the 3D path planning problem, including genetic algorithm, memetic algorithm, particle swarm optimization, ant colony optimization, and shuffled frog leaping algorithm. Zhu et al. [17] proposed an improved self-organizing map and velocity synthesis method for multi-AUV path planning in a 3D underwater workspace. Those methods above have their own advantages; however, there are some shortcomings that should be studied further. For example, the effects of the vision-based methods are not very good in the underwater environment; the computing of some evolution based methods is complex; and the methods based on the assumption that the underwater environments are completely known are not suitable for the actual situation.

To deal with those problems introduced above, some improved and novel methods have been proposed for the real-time path planning of AUVs. For example, Yuan and Qu [18] designed an optimal real-time collision-free trajectory for AUV in a 3D unknown underwater space. In their algorithm, the 3D motion planning problem was reduced to a 2D problem which can greatly reduce the computational efforts. Acosta et al. [19] proposed a knowledge-based approach for an AUV path planner development, by developing a real-time expert system. Saravanakumar and Asokan [20] presented a multipoint potential field method, and a simple strategy was used to avoid the local minima in 3D space. Because of the complexity of real-time path planning for AUV in 3D underwater environments, the traditional methods have some technological bottlenecks. For example, the fuzzy logic based methods cannot find all the fuzzy rules easily and the general neural network based methods often need a learning process, which are not suitable for real-time path planning.

Thus, more and more researchers are focusing on the bioinspired methods [21, 22]. Bioinspired intelligent methods are of a new type with more lifelike working mechanisms to an individual or a group of organisms, which usually have higher efficiency than the traditional artificial intelligent algorithms [23]. Among these bioinspired intelligent methods, a bioinspired neural network (BINN) has been developed to deal with the real-time path planning problem [24–26]. This BINN is inspired from the membrane model for a biological neural system [27] and the shunting model [28]. It is topologically organized which does not need any learning process. Therefore, it can work in real-time; namely, the robot motion planner responds immediately to the dynamic environment. Furthermore, this BINN model is not sensitive to any irrelevant obstacles or sensor noise for its work mechanism [29]. However, this bioinspired intelligent method has some problems in nature, such as the complex computation and the inefficient use of the target information. To deal with these problems, an improved BINN based real-time path planning method is proposed for AUVs in this paper. In the proposed approach, the target information is used directly in the activities of neurons to accelerate the transfer process of the target information. In addition, to reduce the size of the BINN, a dynamic model is proposed, where the AUV is regarded as the core of BINN and the size of BINN is based on the detection range of sensors. At last, some simulation experiments were conducted, and the experimental results show the efficiency of the proposed approach.

The main contributions of this paper are summarized as follows. (1) The BINN model is dynamic, where the BINN will be reconstructed with the movement of the AUV. Then the working environment of AUV can be very big, which will not affect the computation of the BINN based real-time path planning method. (2) The effect of the target information is enhanced, by introducing a target attractor concept, which can improve the computing efficiency. (3) The real-time performance is improved, using a virtual target concept combined with the proposed dynamic BINN model, which can reduce the negative effect of the small sensor detection range for big-size obstacles. (4) Some simulation experiments were conducted in 3D underwater environments, where the real underwater environments were simulated such as the complex seabed and dynamic obstacles in the ocean.

This paper is organized as follows.  Section 2 presents the improved dynamic bioinspired neural network based path planning approach for AUVs. The simulation experiments for various situations are given in Section 3. In Section 4, some performances of the proposed approach are discussed in detail. Finally, conclusion is given in Section 5.

## 2. Proposed Approach

In this paper, the real-time path planning problem for AUV in a 3D unknown underwater environment was studied. In the studied path planning task, the AUVs have no knowledge about the environment, except the location of the target. The AUVs are equipped with various sensors to detect the environment in a limited range. So, in a way, the working environment is also unknown to the AUVs. In this study, the underwater environment model is presented by a discrete 3D grid map [30, 31], which is labeled as *Ω*. Then the whole work space of the AUV is discretized to cells with the same size, and the environment can be defined as a set of *X∗Y∗Z* map. The cell (denoted by *p*) will be marked as an obstacle if it is occupied; otherwise, it will be marked as a free one. The task of the real-time path planning is to generate an optimal or suboptimal path for the AUV quickly and safely, from the start location to the target location with the movement of the AUV. To complete this task efficiently, an improved BINN based method is proposed. The main reason to use this BINN is that it does not need a learning process which is very suitable for real-time path planning. The details of the general process of the BINN based robot path planning method can be seen in some related literatures, such as [24, 32, 33]; here only the improvements of the proposed BINN based method are introduced in detail as follows.

### 2.1. Dynamic BINN Model

**Figure 1:**
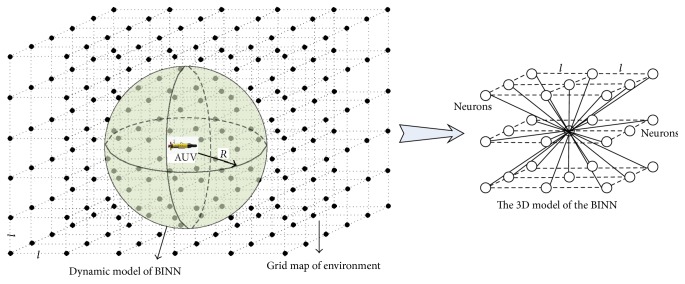
The 3D model of the proposed BINN.

In the general BINN model, each neuron (denoted by *q*) is corresponded to a discrete point of the environment; namely, the whole neural network will cover the whole environment. So the computing time and energy cost will be very large when the working environment is very big. When the BINN model is used for an AUV working in a 3D environment, the number of neurons will be very big and the computing efficiency of the neural network will be reduced. To deal with this problem, a dynamic model is proposed, where the AUV is regarded as the core of the BINN model and the neural network is reconstructed with the movement of the AUV. A schematic map of this dynamic model is shown in Figure 1. The size of the BINN model is set as the maximum detection radius *R* of the onboard sensors; namely, the distance from every neuron *q*_*i*_ in the proposed dynamic BINN model to the core of the BINN *q*_*c*_ (the neuron at the position of the AUV) should be satisfied by

(1) $$\begin{array}{c} 0\leq D\left(\;q_{i},q_{c}\;\right)\leq R, \end{array}$$

where D(·) is the function to calculate the distance of two positions in the 3D space:

(2) $$\begin{array}{c} D\left(\;p_{i},p_{j}\;\right)=\sqrt{{\left(x_{i}-x_{j}\right)}^{2}+{\left(y_{i}-y_{j}\right)}^{2}+{\left(z_{i}-z_{j}\right)}^{2}}, \end{array}$$

where (*x*_*i*_, *y*_*i*_, *z*_*i*_) and (*x*_*j*_, *y*_*j*_, *z*_*j*_) are the coordinates of the *i*th and the *j*th positions, respectively.

**Remark 1 .**

To easily realize the proposed dynamic BINN based path planning method, the discretized dimension of the environment is set the same as the BINN neural network in this study (see Figure 1). To reduce the number of the neurons, the discretized dimension of the environment should be set as big as possible; however, if the discretized dimension of the environment is too big, the generated path cannot be smooth enough. Thus, the distance *l* of two neighboring neurons is set the same as one step of the AUV; namely,

(3) $$\begin{array}{c} l=V_{a}*S_{t}, \end{array}$$

where *V*_*a*_ is the velocity of the AUV and *S*_*t*_ is one unit of the time.

**Figure 2:**
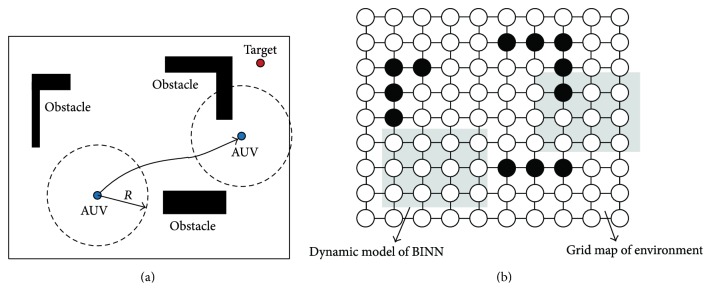
Dynamic working mechanism of the proposed BINN in a 2D space: (a) in the real working environment; (b) in the grid map of environment.

To further introduce this dynamic BINN model, an example of this neural network working in a 2D environment is shown in Figure 2. From Figure 2, we can find that the size of dynamic BINN model is much smaller than the environment, and no matter how large the environment is, the size of the neuron network is fixed and small. With the movement of the AUV, there is only a small part of neurons needed to update their activities at one time, which can reduce the computation cost and energy, especially when the environment is large.

### 2.2. Virtual Target Selection

In the real-time path planning task, the location of the target is known to the AUV at the beginning, but the environment is dynamic and unknown. So the AUV should detect the environment and generate a path in real-time. Because the working environment is often very big, it is impossible for a method to calculate a path for an AUV considering the whole environment. Thus, a dynamic BINN model is proposed; however, the target information cannot be used directly into this dynamic model because the detection range of the AUV's onboard sensor is limited and the environment is unknown. The path will be repeated if there are big-size obstacles (whose size is bigger than the sensor's detection range) on the planned route, because AUV does not know which directions should be used based on the general path planning method. To deal with these problems, a virtual target concept is introduced into the proposed path planning method.

The basic principles to select the virtual target are summarized as follows. (1) The virtual target must be located on the boundary of the dynamic BINN model. (2) The virtual target should be close to the real target as much as possible, which can make the generated path short to the greatest extent. (3) The virtual target should be accessible by the AUV based on the current BINN. (4) There are not any obstacles in the forward direction from AUV to the virtual target. The virtual target is selected based on the following concrete rules:

(4) pv=pn ∣ ∀pk∈H,  Dpn,po≤Dpk,po,  pn∈H,

where *p*_*v*_ and *p*_*o*_ are the cells at the positions of the virtual target and the real target, respectively; *p*_*k*_ denotes a cell of the grid cell set; *p*_*n*_ is the nearest cell to the real target; and *H* is a set of grid cells which should be satisfied by

(5) H=pk ∣ Fpk=0∩0<Dpk,pa≤R∩Opa,pk,po=0,

where *p*_*a*_ is the cell at the position of AUV; F(·) is a function to judge whether the cell is occupied by obstacles, where 0 means free cell and 1 means occupied by an obstacle; and O(·) is a function to judge whether there are any obstacles in the forward direction from AUV to the virtual target.

**Figure 3:**
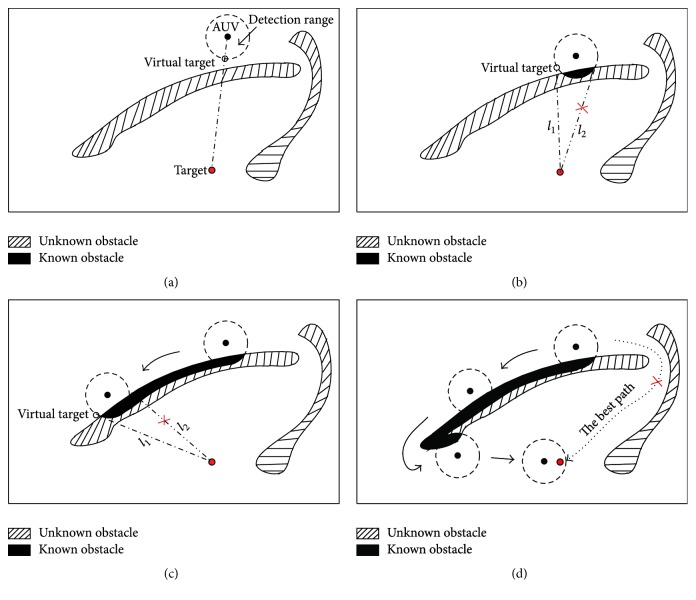
Schematic diagram of virtual target selection.

There are different conditions in the virtual target selection. To illustrate the virtual target selection rules clearly, one example of these conditions is shown in Figure 3 by a 2D environment. For example, in Figure 3(a), there is nothing in the detection range of the AUV; then the neuron closest to the target is selected as the virtual target. In the situation of Figure 3(c), if the virtual target is selected only according to the distance from the neuron to the real target, the path will become repeated. Therefore, the history information of environment should be used. Namely, the neuron which is free and closest to the real target and satisfies the demand that there are no obstacles in the line between this neuron and the real target will be selected as the virtual target.

**Remark 2 .**

The path generated by the proposed method based on the proposed virtual target selection method may not be the best one (see Figure 3(d)), but it is the optimal path for the AUV in the unknown environment, because the detection range of the AUV is limited.

The pseudo-code of the working process of the virtual target selection in this paper is summarized in Algorithm 1.

### 2.3. Target Attractor

After the dynamic model is constructed and the virtual target is selected, a path from the current position of AUV to the virtual target can be generated based on the shunting model [24, 34]:

(6) dxidt=−Axi−D+xiIi−+B−xiIi++∑j=1kwijxj+,

where *x*_*i*_ is the neural activity of the *i*th neuron; *A*, *B*, and *D* are nonnegative constants representing the passive rate and the upper and lower bounds of the neuron activity respectively; *k* is the number of neural connections of the *i*th neuron to its neighboring neurons within the receptive field. In the dynamic 3D BINN model, there are at least 26 neighbors of one neuron (see Figure 1); *w*_*ij*_ is the lateral connection weight from the *i*th neuron to the *j*th neuron, which is a function of the distance (see [24] for details); and [*I*_*i*_]^+^ and [*I*_*i*_]^−^ represent the excitatory and inhibitory inputs to the neuron, where the target and the surrounding positive neurons are the excitatory input and the obstacles mean the inhibitory input. Function [*x*]^+^ is a linear-above-threshold function defined as [*x*]^+^ = max⁡{*x*, 0}, and [*x*]^−^ is defined as [*x*]^−^ = max⁡{−*x*, 0}.

With the activity updating continually, the target globally attracts the whole state space of the BINN and the obstacle always keeps its corresponding neuron activity in a very low level. The AUV always pick the best neighboring neuron with the biggest activity as the next position. The selection rule is as follows:

(7) $$\begin{array}{c} q_{n}⟸x_{q_{n}}=\max\left\{\;x_{j},\;j=1,2,\ldots ,k\;\right\}, \end{array}$$

where *x*_*j*_ is the activity of all the neighboring neurons; *q*_*n*_ is the location of the neuron, with the maximum activity in these neurons. As shown in the activity updating equation (6), the target information will be transferred to the position of AUV by the activity of the neurons; then a path with rising activity value will be established. But by this way, the computing efficiency of activity will be very low. To accelerate the target information transfer speed in the neural network, a concept of target attractor is introduced and the general shunting model is modified by

**Figure 4:**
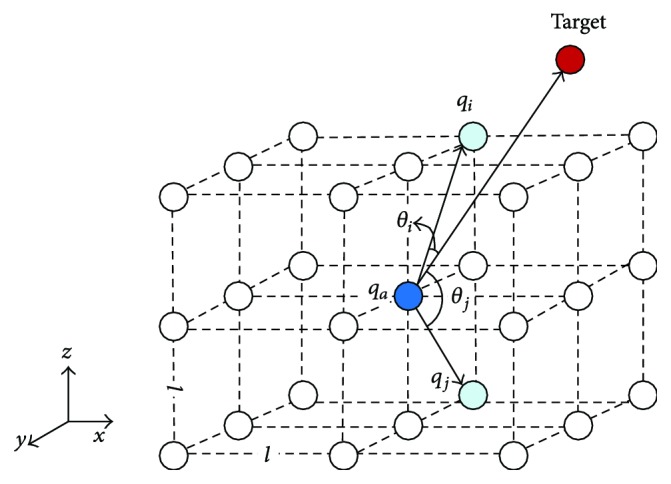
The target attractor concept in the shunting equation.

(8) dxidt=−Axi−D+xiIi−+B−xiIi++∑jkwijxj++ξicos⁡θi,

where *ξ*_*i*_cos⁡*θ*_*i*_ is the proposed target attractor. *ξ*_*i*_ is the weight of the excitatory connection from *q*_*a*_ to *q*_*i*_, and *q*_*a*_ represents the neuron on the position of the AUV. Then the target attractor is defined as

(9) $$\begin{array}{c} \xi_{i}=\frac{\beta }{\left|q_{a}-q_{i}\right|}, \end{array}$$

where *β* is a positive constant no more than 1 and |*q*_*a*_ − *q*_*i*_| represents the Euclidean distance between *q*_*a*_ and *q*_*i*_; *θ*_*i*_ is an angle between two lines (one is from *q*_*a*_ to *q*_*i*_, and the other is from *q*_*a*_ to the target; see Figure 4), which is a variable within [0, *π*] in this study. From the overall view of all the surrounding neurons of *q*_*a*_, we can see that if a neuron is closer to the target, the angle *θ*_*i*_ is smaller and the value of |*q*_*a*_ − *q*_*i*_| is lower.

In the improved shunting equation, the excitatory inputs include [*I*_*i*_]^+^, ∑_*j*=1_^*n*^*w*_*ij*_[*x*_*j*_]^+^ and *ξ*_*i*_cos⁡*θ*_*i*_. Here, the target is the most critical factor, which will affect the activity distribution in two ways. In one way, the target globally influences the whole state space directly because it attracts the AUV wherever the AUV is by the term *ξ*_*i*_cos⁡*θ*_*i*_. On the other way, the positive neural activities derived from the target neuron propagate to the whole space by the term ∑_*j*=1_^*n*^*w*_*ij*_[*x*_*j*_]^+^. Thus, the newly added term speeds up the neuron activity convergence rate, and on the other hand, by establishing direct relation between the AUV and the target, the proposed method can make the path shorter because of the straightforward attraction from the AUV to the target and will not conflict with the obstacles for its weight is much smaller than the weights of obstacles.

The work flow of the whole proposed approach is summarized as follows.

(1) The underwater model is established at first, and the AUV makes sure regarding its own position and the target position.

(2) As the AUV moves, the sensor detection always operates to distinguish the obstacle or clear space in its range.

(3) The neural network and the virtual target change dynamically, and the dynamic activity landscape of the neural network is updated by (8).

(4) When the target is within the range of the AUV, the real target position will replace the virtual target.

(5) When the AUV gets the target position, the task is successfully achieved.

## 3. Simulation Experiments

To prove the effectiveness of the proposed approach for real-time path planning in 3D unknown underwater environments, various simulation experiments were conducted at the platform of MATLAB. When the AUV is working in the water, there are two main conditions: one condition is that the target is floating in the middle of the water and the other is that the target is on the seabed. So both the two conditions were simulated with different cases in this paper, including that there were some moving obstacles in the water. In addition, two experiments were conducted considering the cases having more challenging tasks. In order to easily realize the simulation experiments, the AUV was assumed as a point without any shape, and the signal gain and noise modulation were not considered in this study. The parameters in all the experiments are the same and listed in Tables 1 and 2. The AUV is denoted by blue solid circles and the target is represented by red triangle. To make the generated path of the experiments easy to understand, the path planning process in different stages will be shown in different points of view. In this paper, the view angle is denoted by

(10) $$\begin{array}{c} View=\left(\;az,el\;\right), \end{array}$$

where *az* means azimuth and *el* means elevation of the 3D experimental results.

### 3.1. The Target Is Floating in the Middle of Water

To test the performance of the proposed path planning method for AUV, these experiments were conducted where the target was floating in the middle of water. In the water, there are some obstacles, such as the relative static obstacles (for example, the floating garbage and shipwreck) and the moving obstacles (for example, fish and other AUVs). Thus, two different experiments were conducted.

#### 3.1.1. In Static Environment

**Figure 5:**
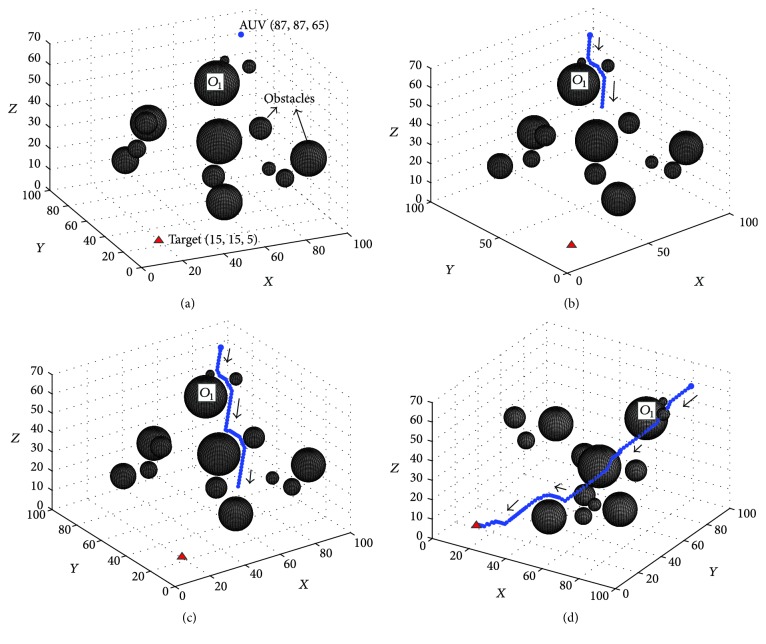
The results of the path planning experiment in static environment: (a) Step = 0, View = (−24°, 22°); (b) Step = 28, View = (−40°, 26°); (c) Step = 59, View = (−35°, 26°); (d) Step = 100, View = (−32°, 26°).

**Figure 6:**
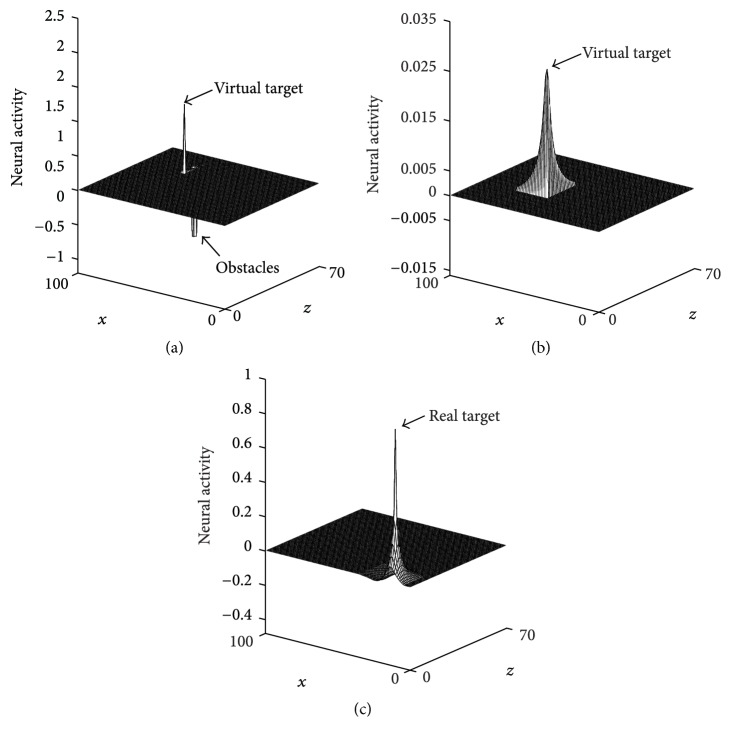
The dynamic activity landscapes of three profiles from *y*-axis during the experiment in static environment: (a) Step = 10, *Y* = 67; (b) Step = 50, *Y* = 31; (c) Step = 100, *Y* = 15.

To demonstrate the basic performance of the proposed approach, this experiment was conducted, where the environment was static and full of obstacles with various sizes (see Figure 5(a)). The initial position of AUV and the target are (87,87,65) and (15,15,5), respectively. The real-time path planning results of this experiment are shown in Figures 5(b)to 5(d). The dynamic activity landscapes of three profiles from *Y*-axis are shown in Figure 6.

The results in Figure 5 show that the proposed approach can find a relative smooth path for the AUV to the target in the middle of the water. The AUV can avoid the obstacles automatically based on the generated path. At the first time, the AUV knows nothing about the obstacles, so it goes straight to the target position until it detects the obstacle *O*_1_; then the AUV successfully avoids the obstacle and goes straight to the target continually (see Figure 5(b)). With the movement of the AUV, the proposed BINN based method can generate an appropriate virtual target for the AUV, which can make the AUV go to the target in an optimal and safe path (see Figures 5(c) and 5(d)). The results in Figure 6 show that the neural network can move with the AUV, and the neural activity is largest at the position of the virtual and real target but smallest at that of obstacles.

#### 3.1.2. In Dynamic Environment

**Figure 7:**
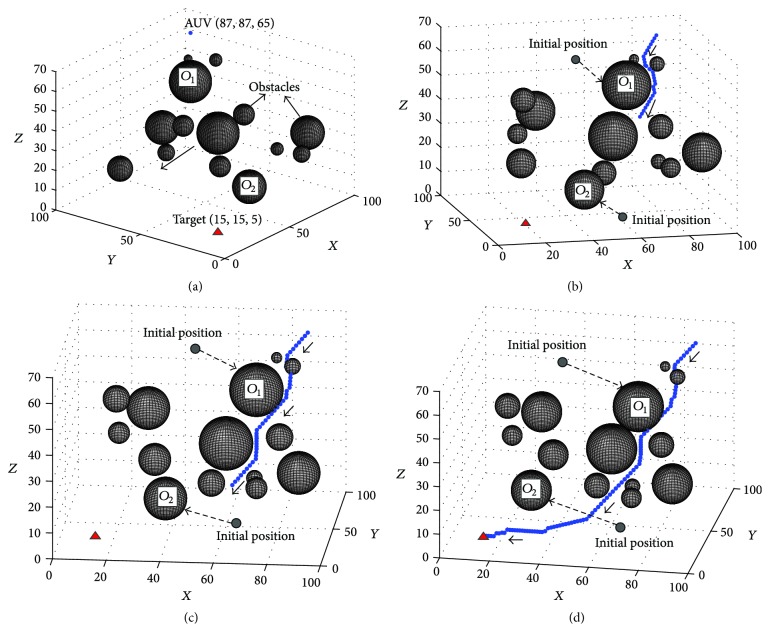
The results of the path planning experiment in dynamic environment: (a) Step = 0, View = (−49°, 30°); (b) Step = 32, View = (−45°, 34°); (c) Step = 54, View = (12°, 20°); (d) Step = 99, View = (14°, 20°).

To test the performance of the proposed approach in dynamic environments, this experiment was conducted, where the initial environment was the same as that of the static experiment (see Figure 7(a)), expect that the obstacles *O*_1_ and *O*_2_ would move randomly while the AUV was moving towards the target. Compared to the environment in Figure 5(a), the obstacle *O*_1_ was moving to block the way of AUV to the target, but the obstacle *O*_2_ was moving away from the path of AUV. The path planning results in the dynamic environment are shown in Figures 7(b)to 7(d).

The results in Figure 7 show that the proposed dynamic BINN based path planning method can generate the path in real-time. The main reason is that the proposed BINN model can move with the movements of the AUV, so if the AUV detects an obstacle moving into its detection range, the path will be replanned immediately (see Figure 7(b)). This characteristic is very important for AUV path planning in the dynamic underwater environment. When the obstacle goes out of the detection range, the AUV can go back to the optimal path again, which can keep the whole path optimal and safe for the AUV (see Figure 7(d)).

### 3.2. The Target Is Located at the Seabed

In the AUV path planning task, sometimes the target may be at the seabed. The seabed environment was very different from that of the middle of water. There are some big underwater mountains or deep valleys, which will make the path planning more difficult when the target is located in the seabed. To test the performance of the proposed method in these situations, some experiments were conducted.

#### 3.2.1. Behind Underwater Mountain

**Figure 8:**
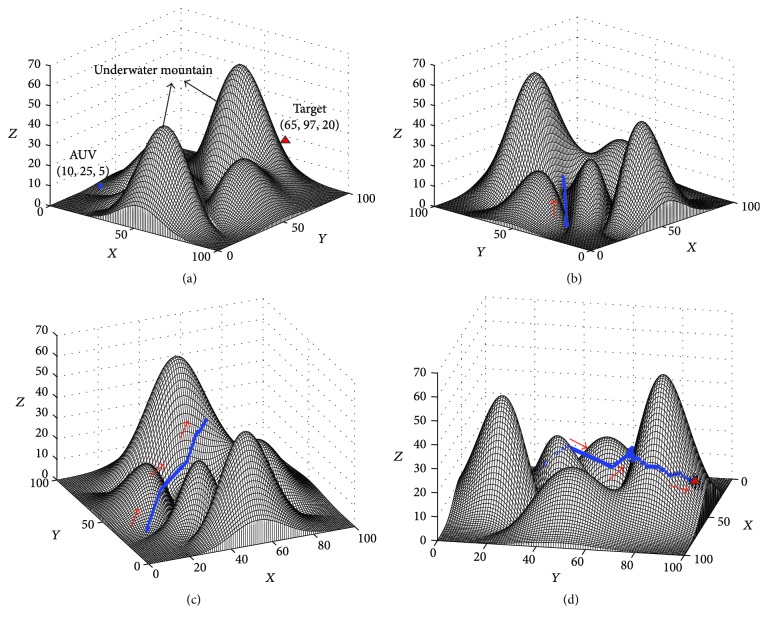
The results of the path planning experiment when target is behind the underwater mountain: (a) Step = 0, View = (38°, 20°); (b) Step = 20, View = (48°, 18°); (c) Step = 50, View = (−24°, 24°); (d) Step = 82, View = (101°, 18°).

In this experiment, the target was located on a big underwater mountain, and the AUV was in front of this mountain. The environment of this experiment is shown in Figure 8(a), where the positions of the AUV and the target are (10,25,5) and (65,97,20), respectively. The path planning results in this experiment are shown in Figures 8(b)to 8(d).

The results in Figure 8 show that the AUV still can find a path to the target efficiently, when the target is located behind an underwater mountain that is bigger than the detection range of the sensors. The AUV can go directly to the mountain, behind which the target is located. When the AUV detects that there is a very big obstacle, the adaptive virtual target is calculated, which makes the AUV move around the mountain successfully (see Figure 8(b)). The final path for AUV in this experiment shows that the proposed approach can deal with the path planning of big obstacles effectively (see Figure 8(d)).

#### 3.2.2. In Deep Underwater Valley

**Figure 9:**
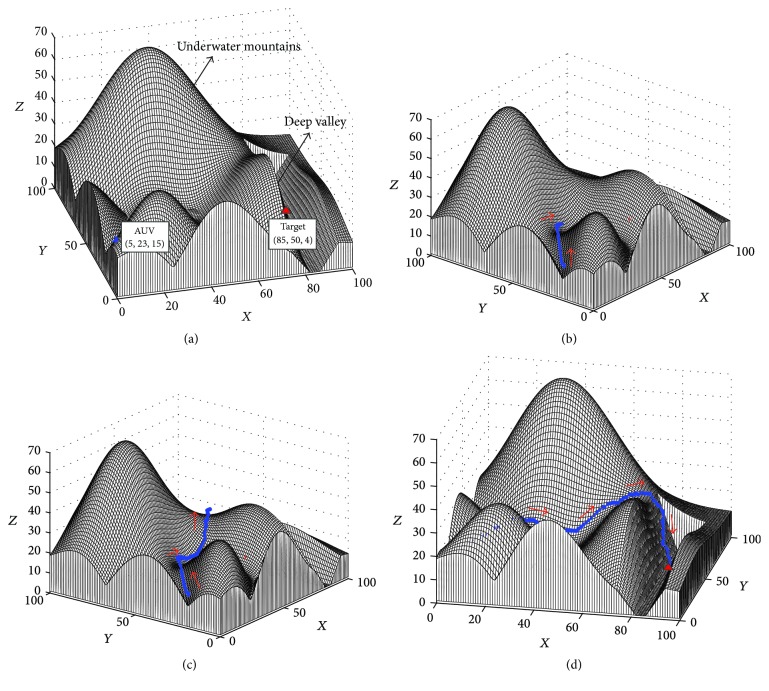
The results of the path planning experiment when target is in deep underwater valley: (a) Step = 0, View = (−15°, 28°); (b) Step = 30, View = (−50°, 24°); (c) Step = 66, View = (−58°, 20°); (d) Step = 102, View = (12°, 20°).

When the target is located in a deep underwater valley, the path planning task will become very difficult. To further test the performance of the proposed approach in this condition, an experiment was conducted, where the initial positions of the AUV and target are shown in Figure 9(a). The positions of the AUV and the target are (5,23,15) and (85,50,4), respectively. The path planning results in this experiment are shown in Figures 9(b)to 9(d).

The results in this experiment show that the proposed approach can generate a path for the AUV from the start position to the target staying in a valley surrounded by highlands without collision with the hills (see Figure 9(b)).

### 3.3. The Tasks Are More Challenging

To further test the effectiveness of the proposed approach in some challenging tasks, two experiments were conducted where the target was dynamic, or there was an underwater cave between the AUV and the target.

#### 3.3.1. The Target Is Dynamic

**Figure 10:**
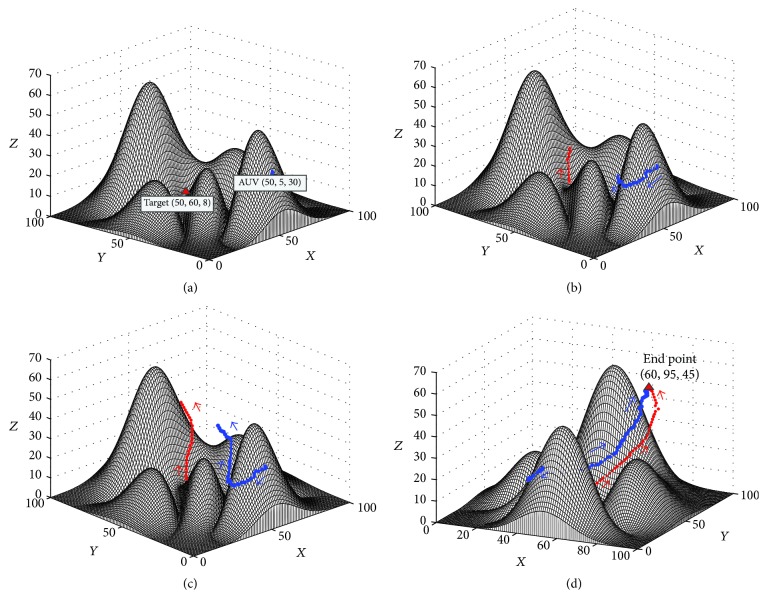
The experimental result of the path planning for a dynamic target: (a) Step = 0, View = (−49°, 18°); (b) Step = 19, View = (−51°, 18°); (c) Step = 54, View = (−43°, 22°), (d) Step = 94, View = (26°, 16°).

To illustrate the performance of the proposed approach in the real-time path planning where the target position will change because of the ocean current or other reasons [35], an experiment was conducted, where the environment was the same as the experiment in Section 3.2.1 (see Figure 8(a)). All the parameters and assumptions of this experiment were the same as those in Section 3.2.1, except that the target would randomly move at a relative low speed (which was set as *V*_*s*_ = 0.3 m/s in this study). The initial positions of the AUV and the target are (50,5,30) and (50,60,8), respectively, which is shown in Figure 10(a). The results of this experiment are shown in Figures 10(b)to 10(d). With the movements of the target, the AUV continuously adjusts its destination and its virtual target (see Figure 10(c)). Finally, the AUV catches the target at the position (60,95,45) shown in Figure 10(d). The results of this experiments show that the proposed approach can regenerate a path for AUV automatically when the position of target changes, which is a very important performance for the path planning of AUV in the sea.

#### 3.3.2. There Is an Underwater Cave between the AUV and the Target

**Figure 11:**
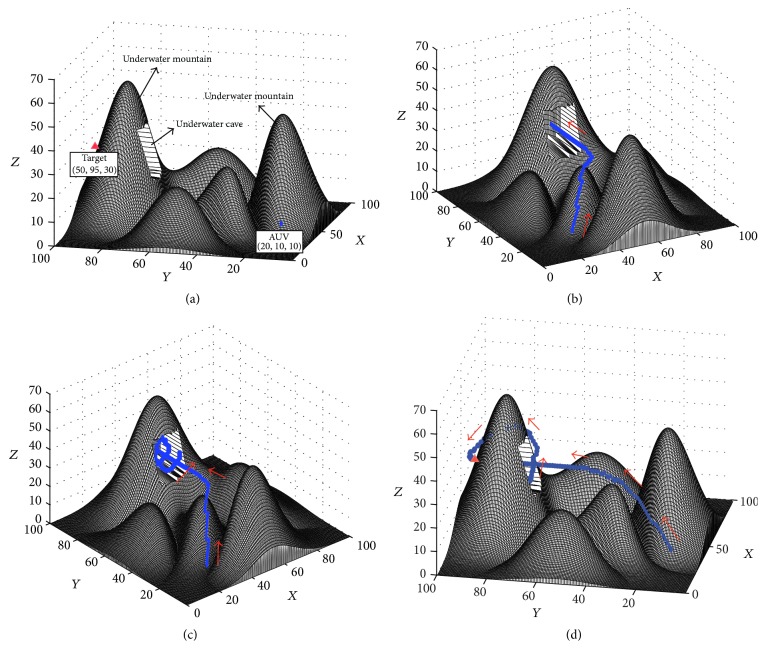
The path planning result of the experiment where an underwater cave exits between the AUV and the target: (a) Step = 0, View = (−76°, 14°); (b) Step = 73, View = (−29°, 23°); (c) Step = 134, View = (−40°, 30°); (d) Step = 184, View = (−79°, 22°).

To further test the performance of the proposed approach in some special underwater environment, a simulation experiment was conducted, where an underwater cave existed between the AUV and the target (see Figure 11(a)). The result of this experiment is shown in Figure 11. The movement of the AUV in this experiment shows that the AUV will come into the cave firstly because there is not any prior knowledge of the environment. However, the AUV can escape from the cave and reach the target based on the proposed dynamic BINN approach (see Figures 11(b)to 11(d)). Although the path is not the shortest one to the target, the performance of the proposed approach is good in this challenging task.

## 4. Discussions

The results of the simulation experiments in Section 3 show that the presented path planning algorithm can deal with the path planning problem in 3D unknown underwater environments effectively. The performances and improvements of the proposed approach are discussed in this section.

**Figure 12:**
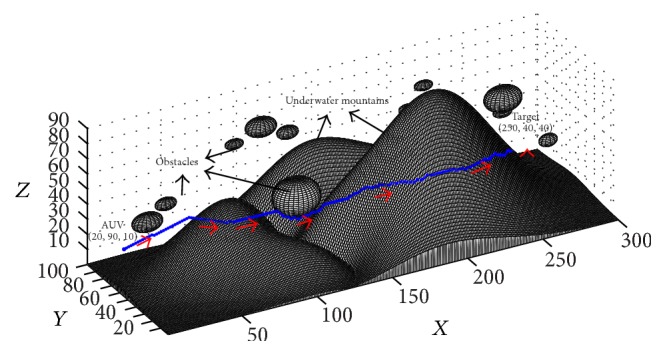
The path planning result of the experiment in a very large underwater environment (View = (−30°, 18°)).

The main improvement of the proposed approach is the computational efficiency, so the performance of the proposed real-time path planning approach in a very large underwater environment is discussed firstly. Then a simulation experiment was conducted, where the parameters of the proposed approach were the same as those in Section 3, except that the environment was bigger (having dimensions 300*∗*100*∗*100) and the distribution of the obstacles was more complex. The initial position is (20,90,10) and the target position is (250,40,40). The final path generated by the proposed approach is shown in Figure 12. The result in Figure 12 shows that the proposed approach can deal with the path planning for AUV in a very large environment efficiently. To show the performance of the proposed approach in this large environment, the computation time in this task is compared with those of the experiments in Section 3 (see Table 3). The results in Table 3 show that the computation time of the proposed dynamic BINN based approach almost linearly depends on the path length and the performance of the proposed approach has not been obviously affected by the size of the environment. This performance is better than that of the general BINN model based method which needs to calculate activities of all the neurons in the whole environment (see [24, 29]). In addition, the results of seven experiments in Table 3 show that the computation efficiency of the proposed approach is very good, which is very important for the real-time path planning task.

The virtual target selection strategy introduced in Section 2.2 and the simulation results based on the proposed approach show that the repeated path problem is solved efficiently. In the general BINN based method, the robot will be trapped in some difficult environment when the environment is unknown and the sensor's range is limited while the obstacle is very big, such as the big mountain in Section 3.2.1 and the underwater cave in Section 3.3.2. However, the proposed approach can deal with these challenging problems based on the virtual target selection strategy, where the history information of environment is used fully and there should be no any obstacles in the forward direction from AUV to the virtual target (see Figure 3). This performance of the proposed method is better than those of the traditional artificial potential field methods and other optimization methods which will encounter the local minimum problem [20, 36].

**Figure 13:**
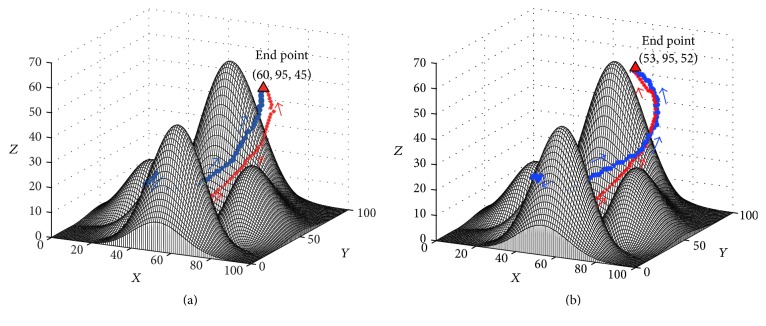
The comparison experiment results for a dynamic target: (a) based on the proposed approach; (b) based on the method without the target attractor.

Another improvement of the proposed approach is the target attractor in the computation of the neuron activity (see (6)), which will be discussed in the end part. A comparison experiment was conducted, where the proposed approach was compared with a method which had the same parameters and work flow as the proposed approach, except that the target attractor was removed from (6). The experiment in Section 3.3.1 is used as reference. The experimental results are shown in Figure 13 and Table 4. The results of this comparison experiment show that the AUV reaches the target at (60,95,45) by 94 steps based on the proposed approach, but the AUV reaches the target at (53,95,52) by 98 steps based on the method without the target attractor. The reason is that the target attractor can increase the computation efficiency for the neuron activity, where the efficiency is increased more than 3 times using the target attractor (see Table 4). The results show that the target attractor of the proposed approach can improve the real-time performance of the BINN based path planning method. On the other hand, the target attractor directly attracts the AUV to the target and the AUV is more capable of adapting to the changes in the target trajectory than without the target attractor, which helps the AUV get to the target quicker and the generated path shorter (see Figure 13 and Table 4). This performance factor is very useful in the path planning task for dynamic target.

As the discussions above and the work process introduced in Section 2, several points about the proposed BINN based approach are worth noticing. (1) This model is originally derived from Hodgkin and Huxley's [27] biological membrane model, which is biologically plausible. (2) The proposed BINN is topologically organized, and the robot motion is planned based on the dynamic activity landscape of the neural network, which needs no learning process. (3) Because the computational complexity of the general BINN linearly depends on the number of the neurons in the neural network, it is not suitable for a very complicated and large environment. In the proposed approach, this problem is resolved efficiently. (4) The computation of the proposed BINN based approach is simpler than other optimization methods, such as GA and PSO based methods [37]. In addition, the proposed BINN based method is distinct from the previous path planning methods based on the general BINN model, where the neural network is static and only the distance information of the target is used [13, 24, 38].

## 5. Conclusion

Real-time path planning for an AUV in 3D underwater environments has been investigated in this paper. A dynamic bioinspired neural network is proposed, where a virtual target strategy is used to help the AUV find an optimal or suboptimal path from the start position to the target efficiently. In the proposed BINN based method, a target attractor is introduced into the neuron activity updating equation, to improve the computing efficiency of the bioinspired neural network for real-time path planning. The proposed approach can deal with the path planning problem in various situations: that is, the target is floating in the water and present at the seabed, the environment is large, and the position of the target is changing. However, there are still some limitations in the BINN based path planning approach which should be further studied: that is, no any prior knowledge of the environment or the task can be used, which makes the whole efficiency low. To deal with this problem, some learning based methods such as reinforcement learning method may be fused into the BINN based method to guarantee the real-time performance and take full advantage of the prior information. In future work the real experiments for AUV path planning will be conducted and some new bioinspired methods will be studied to improve the path planning efficiency.

## Figures and Tables

**Algorithm 1:**
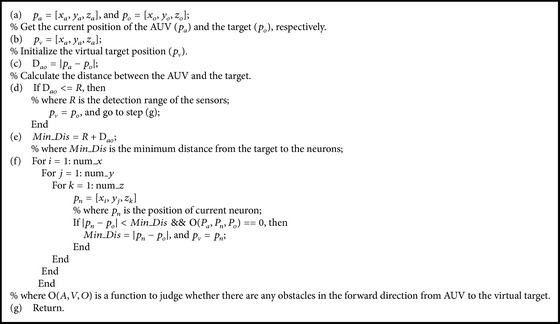
Pseudo-code of virtual target selection algorithm.

**Table 1:** Parameters of the proposed BINN model.

Parameter	Value	Remark
*A*	15	The passive decay rate of the neural activity
*B*	1	The upper bound of the neural network
*D*	1	The lower bound of the neural network
*β*	1	The weight of direct attraction from the target

**Table 2:** Parameters of the experimental settings.

Parameter	Value	Remark
*X*	100 m	The length of the environment model
*Y*	100 m	The weight of the environment model
*Z*	70 m	The height of the environment model
*R*	10 m	The detection range of the sensors
*V*_*a*_	1 m/s	The velocity of the AUV
*V*_*b*_	0.5 m/s	The velocity of the dynamic obstacles

**Table 3:** Comparison on the computing time of different experiments.

Experiment	Environment	Length of path	Total steps	Total time	Efficiency
Experiment in Figure 5	100*∗*100*∗*70	140.83 m	100	85.32 s	0.85
Experiment in Figure 7	100*∗*100*∗*70	139.61 m	99	84.89 s	0.86
Experiment in Figure 8	100*∗*100*∗*70	119.78 m	82	67.78 s	0.83
Experiment in Figure 9	100*∗*100*∗*70	132.24 m	102	87.06 s	0.85
Experiment in Figure 10	100*∗*100*∗*70	145.41 m	94	77.76 s	0.83
Experiment in Figure 11	100*∗*100*∗*70	223.46 m	184	162.56 s	0.88
Experiment in Figure 12	300*∗*100*∗*100	294.41 m	231	205.88 s	0.89

These experiments were conducted in the same computer with 2.9 GHz CPU and 4.0 GB RAM.

The computing efficiency is defined as the ratio of the total computing time to the total steps.

**Table 4:** The comparison experiment on the target attractor.

Experiment	Length of path	Total steps	Total time	Efficiency
Experiment in Figure 13(a)	145.41 m	94	77.76 s	0.83
Experiment in Figure 13(b)	154.62 m	98	269.60 s	2.75
